# P53 Family Members Modulate the Expression of *PRODH*, but Not *PRODH2*, via Intronic p53 Response Elements

**DOI:** 10.1371/journal.pone.0069152

**Published:** 2013-07-08

**Authors:** Ivan Raimondi, Yari Ciribilli, Paola Monti, Alessandra Bisio, Loredano Pollegioni, Gilberto Fronza, Alberto Inga, Paola Campomenosi

**Affiliations:** 1 Department of Biotechnology and Life Sciences, DBSV, University of Insubria, Varese, Italy; 2 Laboratory of Transcriptional Networks, Centre for Integrative Biology, CIBIO, University of Trento, Mattarello, Trento, Italy; 3 Department of Diagnosis, Pathology and Treatment of High Technological Complexity, IRCCS Azienda Ospedaliera Universitaria San Martino – IST - Istituto Nazionale Per La Ricerca Sul Cancro, Genova, Italy; 4 The Protein Factory, Centro Interuniversitario di Ricerca in Biotecnologie Proteiche, Politecnico di Milano, ICRM-CNR Milano and Università degli Studi dell'Insubria, Varese, Italy; University of Saarland Medical School, Germany

## Abstract

The tumor suppressor p53 was previously shown to markedly up-regulate the expression of the *PRODH* gene, encoding the proline dehydrogenase (PRODH) enzyme, which catalyzes the first step in proline degradation. Also PRODH2, which degrades 4-hydroxy-L-proline, a product of protein (e.g. collagen) catabolism, was recently described as a p53 target. Here, we confirmed p53-dependent induction of endogenous *PRODH* in response to genotoxic damage in cell lines of different histological origin. We established that over-expression of TAp73β or TAp63β is sufficient to induce *PRODH* expression in p53-null cells and that *PRODH* expression parallels the modulation of endogenous p73 by genotoxic drugs in several cell lines. The p53, p63, and p73-dependent transcriptional activation was linked to specific intronic response elements (REs), among those predicted by bioinformatics tools and experimentally validated by a yeast-based transactivation assay. p53 occupancy measurements were validated in HCT116 and MCF7 human cell lines. Conversely, *PRODH2* was not responsive to p63 nor p73 and, at best, could be considered a weak p53 target. In fact, minimal levels of PRODH2 transcript induction by genotoxic stress was observed exclusively in one of four p53 wild-type cell lines tested. Consistently, all predicted p53 REs in *PRODH2* were poor matches to the p53 RE consensus and showed very weak responsiveness, only to p53, in the functional assay. Taken together, our results highlight that *PRODH*, but not *PRODH2*, expression is under the control of p53 family members, specifically p53 and p73. This supports a deeper link between proteins of the p53-family and metabolic pathways, as PRODH modulates the balance of proline and glutamate levels and those of their derivative alpha-keto-glutarate (α-KG) under normal and pathological (tumor) conditions.

## Introduction

The p53 protein exerts its tumour suppressive function acting primarily as a transcription factor, that controls the expression of a large and ever increasing number of target genes in response to a variety of stresses [1–3]. Well known outcomes are cell cycle arrest, DNA repair, apoptosis, but more recently p53 involvement in the induction of autophagy and regulation of metabolism has also been described [4,5].

Historically, p53 represents the founder member of the p53 family, to which also p63 and p73 belong. These proteins share the highest level of homology in the DNA binding domain and often recognize the same REs in the promoter of target genes. However, the pattern of transcribed genes upon induction of the different family members does not overlap completely [6] and neither do the biological functions, as exemplified by their different roles during embryonic development [7].

To complicate matters, the various p53 family members can also influence each other’s function and transactivation activity through a complex network created by the different transactivation efficiency of the various isoforms and the presence of “p53” REs in their own regulatory regions [8,9]. Clearly, there is a complex transactivation network within the p53 family and between the p53 family and other transcription factors in the regulation of target genes [10,11]. The canonical p53 RE consists of two decameric half-sites separated by a 0-13 base spacer (n): RRRCWWGYYY-(n)-RRRCWWGYYY (R = purine; W=A/T; Y = pyrimidine) [12]. Moreover, non-canonical binding sites have been identified comprising half-sites and three quarter sites, expanding the universe of potential downstream target genes which may be regulated by p53 [13].

This underlines the importance of understanding how transactivation specificity arises through the mapping and the characterization of the REs present in target genes. Regulatory regions within genes are a complex field of investigation. Thus, their thorough characterization in terms of sequence and location may help assessing coordinated regulation by transcription factors, as well as cell type and cell context (type of stress, kinetics) dependencies on gene expression, ultimately contributing to define gene functions [11,14].

Several years ago Polyak and colleagues identified *PIG6*, also known as *PRODH/POX*, among the apoptotic genes induced by p53 after adriamycin treatment [15]. Nevertheless, a systematic search and validation of the p53 REs in this gene have never been carried out. As a consequence, *PRODH* is not considered as a proven p53 target in most of the published reviews [1,2]. Since its discovery, evidence has been accumulating on the role that proline dehydrogenase, the protein encoded by the *PRODH* gene, could play in suppressing tumorigenesis, suggesting its contribution as an apoptosis effector through ROS induction [16]. Very recently, a PRODH-dependent induction of autophagy has also been described [17]. The biochemical function of PRODH (EC 1.5.99.8) is the oxidation of proline to Δ'-pyrroline-5-carboxylic acid (P5C), which is converted to glutamate by P5C dehydrogenase (EC 1.5.1.12). Notably, also the gene encoding P5C dehydrogenase, *ALDH4*, has been reported to be a target of p53 [18], suggesting the importance of the proline to glutamate conversion in mediating p53 functions.

More recently, some Authors showed that also 4-hydroxy-L-proline (OH-proline) dehydrogenase (EC 1.1.1.104), encoded by the *PRODH2* gene, was induced by p53 [19], a result, however, not confirmed by others [20]. Like proline, OH-proline, the substrate of OH-proline dehydrogenase, is present in some cellular, extracellular and dietary proteins, and represents an abundant source of substrate. While downstream metabolites of proline can impact several aspects of cellular metabolism, OH-proline derivatives compounds can be used to generate ATP or ROS, but do not have anaplerotic or regulatory functions [19].

The aim of this study was to identify and to validate the p53 REs present in the *PRODH* and *PRODH2* genes and to investigate their responsiveness also to the other p53 family members. Here we show that four intronic p53 REs, located in introns 2 and 3 of the *PRODH* gene, are the most active among the REs examined. Interestingly, one of them is efficiently transactivated by all p53 family members. Conversely, the putative REs identified in the *PRODH2* gene respond poorly even in the presence of high p53 levels and are inactive with p63 and p73, as revealed with a yeast functional assay. Moreover, *PRODH2* expression was weakly detectable following genotoxic stress only in one of the p53 wild-type cell lines we tested, consistent with heterogeneous results in the literature [19,20].

## Materials and Methods

### Reagents

Doxorubicin (DOXO) and 5-fluorouracil (5FU) were from Sigma Aldrich (Milan, Italy); Nutlin-3A was purchased from Alexis Biochemicals (Enzo Life Sciences, Exeter, UK). All oligonucleotides were from *Eurofins MWG Operon* (Ebersberg, Germany). Bacteriological reagents (Bactoagar, Yeast extract, Peptone) were from DIFCO (BD Biosciences, Milan, Italy) and all other reagents were from Sigma Aldrich (Milan, Italy).

### Cell lines and treatments

The human breast adenocarcinoma-derived MCF7 and MDA-MB-231 cell lines were obtained from the InterLab Cell Line Collection bank, ICLC (Genoa, Italy); the colon adenocarcinoma HCT116 (p53^+/+^) cell line and its p53^−/−^ derivative were a gift from B. Vogelstein (The Johns Hopkins Kimmel Cancer Center, Baltimore, Maryland, USA) [21]. LoVo colon adenocarcinoma cells were a gift from M. Broggini (Istituto Farmacologico Mario Negri, Milan, Italy) [22]; Rh30 rhabdomyosarcoma cell line was donated by Dr. A. Rosolen (Clinica di Oncoematologia Pediatrica, University of Padua, Italy) [23]; the hepatocellular carcinoma derived Mahlavu and HepG2 cell lines were a generous gift of Dr. M.L. Neri (University of Ferrara, Italy) [24] and A. Provenzani (University of Trento, Italy) [25], respectively. Finally, HaCat immortalized keratinocytes, JHU-011 and JHU-029 head and neck squamous cell carcinoma (HNSCC) cell lines were obtained from the Sidransky lab at Johns Hopkins University (Baltimore, MD, USA) [26–28]. Cells were maintained in DMEM or RPMI supplemented with 10% FCS, 1% glutamine and antibiotics (100 units/ml penicillin plus 100 µg/ml streptomycin) and routinely checked to exclude the presence of mycoplasms.

To study *PRODH* expression in response to endogenous p53 induction or stabilization, or endogenous p63 and p73 modulation, cells were seeded at 80% confluence and treated with genotoxic agents or Nutlin-3A at the indicated concentrations for 16 hours. For transient transfection experiments with HCT116 p53^-/-^, 7 x 10^5^ cells were seeded in 6-well plates 24 hours before transfection to reach ~70% confluence on transfection day. Cells were transfected using 2 µg plasmid DNA/well and the TransIT-LT1 transfection reagent (Mirus, Milan, Italy) according to the manufacturer’s instructions. Human p53 was expressed from the pC53-SN3 plasmid [29], while p63β and p73β cDNAs were expressed from pCDNA3.1 [11]. pCDNA3.1 vector with no insert (empty vector) was used as negative control in each transfection. All mammalian constructs were extracted from XL1blue *E. coli* cells using the endotoxin free PureYield plasmid midi-prep kit, according to the manufacturer’s protocol (Promega, Milan, Italy). In all experiments, cells were harvested 24 hours after transfection, trypsinized and collected for RNA extraction.

### Antibodies and western blotting

Soluble protein extracts were obtained by mechanically scraping the cells from 100 mm plates in PBS containing 5 mM Na _2_EDTA, followed by cell counting and resuspension in RIPA buffer supplemented with protease inhibitors (PMSF, benzamidine, aprotinin, and leupeptin). After 1 hour incubation at 4°C under rotation and centrifugation to remove the insoluble fraction, the proteins’ concentration was evaluated using the Bradford reagent, and a standard curve obtained with bovine serum albumin. Twenty to 150 µg of extract were used for SDS–PAGE, depending on the protein to be immunodetected. Immunoblot analyses were performed using the i-Blot semi-dry system with nitrocellulose membranes (InVitrogen, Life Technologies, Milan, Italy) and detected with the ECL Select chemiluminescent substrate (GE Health Care, Milan, Italy). When necessary, blots were stripped by standard methods and re-probed with the indicated antibody. Mouse monoclonal DO-1 anti-p53 antibody (sc-126) and 4A4 anti-p63 (sc-8431) were from Santa Cruz Biotechnology (Heidelberg, Germany), while mouse monoclonal ER-15 anti-p73 antibody (OP 109) was from Calbiochem-Millipore. Rabbit anti β-actin (A2066) from Sigma (Milan, Italy) or 3F3-G2 mouse monoclonal anti β-tubulin (sc-53140) from Santa Cruz were used for normalization.

### Analysis of *PRODH* and *PRODH2* transcript levels

To quantify *PRODH* and *PRODH2* mRNAs following treatments or transfections, cells were harvested and washed once with PBS. Total RNA was extracted using the RNeasy Kit (Qiagen, Milan, Italy) according to the manufacturer’s instructions. For real-time quantitative PCR (qPCR), cDNA was generated from 2 µg of RNA by using the RevertAid First Strand cDNA Synthesis Kit (Fermentas, Milan, Italy) or the iScript Reverse Transcription Supermix for qPCR (Biorad, Milan, Italy). qPCR was performed on a RotorGene 3000 thermal cycler (Corbett Life Science, Ancona, Italy) or on a StepOne thermal cycler (AB, Milan, Italy) using the KAPA Probe Fast Universal 2X qPCR Master Mix ( *Resnova* , Rome, Italy) with Taqman assays (AB, Milan, Italy) or the Sso Advanced Sybr Green Supermix (Biorad, Milan, Italy). Primers are reported in Table S1. Relative mRNA quantification was obtained using the ΔΔCt method, where the glyceraldehyde 3-phosphate dehydrogenase (*GAPDH*) or the β2-microglobulin (B2-M) genes served as internal control. *P21*, *NOXA*, *PUMA* or *COL18A1* were used as controls for the efficacy of modulation of p53 family members by the specific treatment or transfection.

### Construction of yeast reporter strains and media

Nine different *Saccharomyces cerevisiae* reporter strains were constructed, containing the firefly luciferase gene under the control of the p53 RE found by bioinformatics tools (see below) in the *PRODH* and *PRODH2* genes. To insert the putative p53 RE upstream of the luciferase reporter gene the “delitto perfetto” approach for *in vivo* mutagenesis was used [30], starting from the available master reporter strain yLFM-ICORE. The master strain contains the luciferase cDNA integrated in the yeast genome downstream a minimal promoter derived from the *CYC1* gene. The counter selectable ICORE cassette is located 5’ to the minimal promoter and confers high targeting efficiency of the locus by oligonucleotides that contain the desired RE sequences flanked by appropriate homology regions (Table S2) [30]. The recombinant yeast strains were checked by colony PCR and direct sequencing for proper positioning of the inserted REs (BMR Genomics, Padua, Italy).

Yeast cells were grown in YPDA medium (1% yeast extract, 2% peptone, 2% dextrose with the addition of 200 mg/L adenine). For plating, YPDA medium was supplemented with 2% bactoagar, while selective minimal plates lacking tryptophan or leucine but containing adenine (200 mg/L) and dextrose as carbon source were used to isolate transformant clones with expression vectors for p53 family proteins. 5-Fluoro-orotic acid (FOA) and geneticin (G418) were added to the plates when necessary [30].

### Constructs for the expression of p53 family members in *S. cerevisiae*

To express members of the p53 family in yeast, the pTSG- (*TRP1*) or pLSG- (*LEU2*) based constructs, harbouring respectively p73β and p63β cDNAs (TA isoforms) under the control of the *GAL1,10* inducible promoter, were used [13,31]. This promoter allows to modulate the expression of the proteins under study by varying the galactose concentration in the culture medium The wild-type p53 cDNA was similarly expressed using the pLS89 expression vector (*TRP1*) [31]. Empty vectors pRS-314 or pRS-315 were used as controls; these vectors contain respectively the *TRP1* (as pTSG-) or *LEU2* (as pLSG-) yeast selectable markers.

### Luciferase assays in yeast

To measure the transactivating capacity of p53 family members on the putative p53 REs identified in *PRODH* and *PRODH2* genes, the expression vectors described above were transformed into the yLFM-RE strains using the lithium acetate method. After transformation the yeast strains were grown on minimal medium lacking tryptophan or leucine but containing adenine (200 mg/L) and dextrose as carbon source, to keep the expression of p53 family members inhibited. After 2-3 days at 30°C, transformants were streaked onto the same type of plates and grown for an additional day. For each reporter strain, the basal luciferase activity was measured from the empty vectors pRS314- or pRS315-transformed colonies. As a positive control, the yeast strain carrying one of the p53 REs from the *p21* gene (p21-5’: GAACATGTCC-CAACATGTTG) was used. This strain has been previously described [32].

Transformant colonies were grown in 100 µL of selective medium containing raffinose as the sole carbon source in a transparent 96-well plate for 16-24 hours at 30°C. Different concentrations of galactose (0.008% and 1%) were used to induce low or high levels of expression of the p53 family members. OD_600_ was directly measured in the multi-well plate to normalize for cell density using a multilabel plate reader (Infinite M200-Pro, Tecan, Milan, Italy). Ten µL of cells suspension were transferred to a white 384 plate (BrandTech Scientific Inc., Essex, CT, USA) and mixed with an equal volume of PLB buffer 2X (Passive Lysis Buffer, Promega). After 15 minutes of shaking at room temperature, 10 µL of *Firefly* luciferase substrate (Bright Glo Luciferase Reporter Assay, Promega) were added. Luciferase activity was measured and results were expressed as fold of induction compared to empty vectors.

#### Chromatin immunoprecipitation experiments in HCT116 and MCF7 cell lines

Chromatin immunoprecipitation (ChIP) assays were performed as previously described [33], using the EZ Magna ChIP kit (Upstate Biotechnology, Millipore, Lake Placid, NY, USA). Briefly, MCF7 or HCT116 p53^+/+^ and p53^-/-^ cells were plated onto 150 mm dishes, let to grow for one day and treated with 1.5 µM doxorubicin for 16 hours or left untreated. Cells were then cross-linked with 1% formaldehyde for 10 minutes at 37°C and treated subsequently with 125 mM glycine for 5 minutes. Samples were processed following the manufacturer’s instructions. Cell lysates were then sonicated using conditions that enabled us to evaluate the distinct contribution of the different REs. Sonication was done using a Misonix 4000 instrument equipped with a multiplate horn (Misonix, Qsonica LLC, Newtown, CT, USA). Samples were sonicated using twelve cycles of 20 seconds pulses at 80% of amplitude with a 40 seconds pause in-between and the accuracy of sheared chromatin fragments was checked on a 2% agarose gel. The p53-specific monoclonal antibody DO-1 (Santa Cruz Biotechnology, Milan, Italy) and magnetic Protein G beads were used in the ChIP assay. In MCF7, the mouse IgG (Santa Cruz Biotechnology) was used as background, while the DO-1 binding in the HCT116 p53^-/-^ cell line was considered as background for the occupancy in the HCT116 p53^+/+^ cells. Once reverted the crosslinks, PCR amplifications were performed on immunoprecipitated and purified chromatin using primers to amplify specific regions in the *PRODH* promoter and introns, in the *CCNB1* gene, that does not contain any p53 REs (No Binding Site, NBS: negative control) and in the *P21* gene (positive control) (see Table S1 for a list of oligonucleotide primers used in this work). Furthermore, qPCR was used to quantify the change in site occupancy in DOXO treated compared to untreated samples. The qPCR reaction was performed with 2 µL of each sample and using the Power SYBR® Green PCR Master Mix (Applied Biosystems, Foster City, CA, USA), following manufacturer’s procedures. The results are indicated as percent of input DNA.

### Bioinformatics analysis

Sequences of the human *PRODH* and *PRODH2* reference mRNAs (NM_016335.4 and NM_021232.1, respectively) were retrieved from NCBI (http://www.ncbi.nlm.nih.gov/nucleotide), and the genomic organization was obtained with the UCSC Blat algorithm at http://genome.ucsc.edu/, followed by extension of the promoter region retrieved, using the function “gene sorter” at UCSC.

To search for the p53 REs in the *PRODH* and *PRODH2* genes, the following sites were consulted:

• TESS (http://www.cbil.upenn.edu/tess.);
• p53MH algorithm (http://linkage.rockefeller.edu/ott/p53MH.htm.) [34];
• p53 FamTaG (http://p53famtag.ba.itb.cnr.it/index.php.) [35];
• TFBIND (http://tfbind.hgc.jp/) [36].

## Results

### 1: PRODH levels increase upon genotoxic stress or p53 stabilization

**Figure 1:**
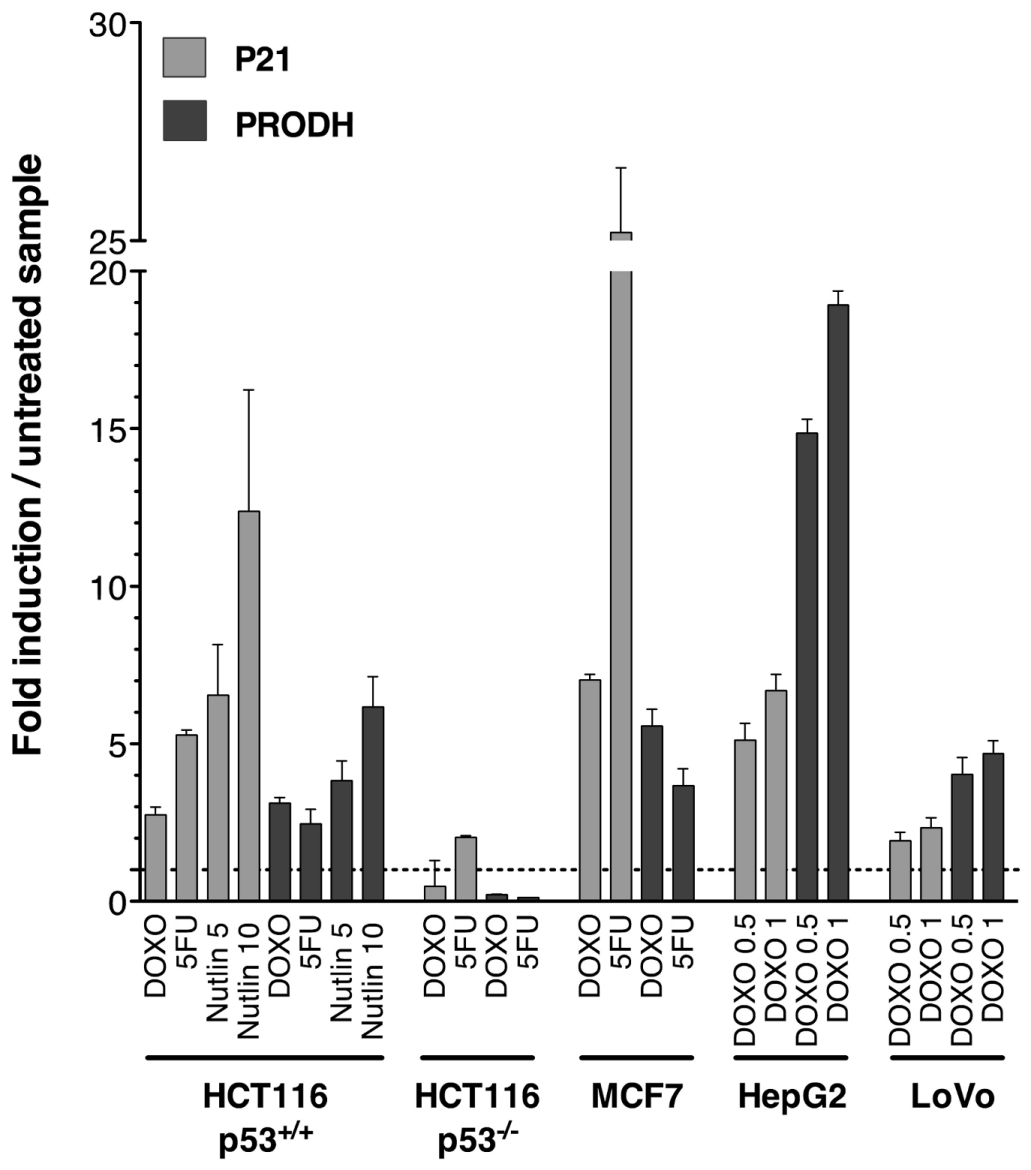
Genotoxic stress and p53 stabilization result in a p53-dependent increase of *PRODH* transcript levels. HCT116 p53^+/+^, HCT116 p53^-/-^, MCF7, HepG2 and LoVo cell lines were treated with the genotoxic compounds Doxorubicin (DOXO, 0.5, 1 or where not indicated, 1.5 µM), 5-Fluorouracil (5FU, 375 µM) or with the p53 stabilizer Nutlin-3A (Nutlin, 5 or 10 µM) for 16 hours before proceeding to total RNA extraction, cDNA preparation and real time q-PCR. The established p53 target gene *P21* is shown for comparison (lighter bars). Bars indicate the average folds of induction obtained in treated *versus* untreated cell samples, plotted together with the standard deviation of three biological replicates. The values obtained in untreated cell lines were set to 1 and shown in the figure as a horizontal broken line.

To confirm that *PRODH* is inducible by genotoxic stress via p53, we treated cell lines of different origin (colon, breast, liver), known to harbour wild-type p53, with DOXO or 5-FU. *PRODH* transcript was readily induced by DOXO or 5-FU in all cell lines tested except in HCT116 p53^-/-^ cells, confirming that induction was indeed p53 dependent (Figure 1). Induction by DOXO was particularly evident in the HepG2 cell line. *PRODH* expression also increased in a dose dependent manner when HCT116 p53^+/+^ cells were treated with the p53 stabilizer Nutlin-3A (Figure 1).

As *PRODH* induction occurred upon increase of p53 levels due to either genotoxic stress or treatment with a p53 stabilizer, we suggest that it is indeed p53 dependent.

### 2: The *PRODH* gene contains numerous putative p53 REs

A combination of four bioinformatic tools for identifying p53 specific or general transcription factors binding sites (p53MH, p53FamTag, Tess, TFBIND) along with manual search was used to scan the *PRODH* gene. Nine putative p53 REs were identified and named according to their distance from the Transcription Start Site (TSS), based on reference sequence NM_016335.4 (Table 1).

**Table 1:** p53 Response elements in the *PRODH* gene.

	Name	Location (bp from TSS)	Mismatches HS 1	Mismatches HS 2	Spacer (bp)	Sequence
1	**-3.1**	Promoter, -3,158	1	2	5	**cGACTTGTCC** *TCAAT* **GAcCAcGCTC**
2	**-0.9**	Promoter, -917	-	1 in ¼ site, 3 in a HS	7, 5	CACC **AGgCT**CCACTAT**GGGCTTGTCT**TCGTG**tGACTTcTgT** *
3	***+*1.7**	Int 2,+1,694	2	1	-	**GGGCAAGgaCGGGCATGCTa**
4	***+*2.8**	Int 2,+2,816	2, 2, 2, 2, 3 in each of the 5 HS		3,0,0,3 bp respectively	**ttACAAGCCC**TAG**GctCATGCCTAGGCATGgTgGctCATGCCT**GTA**AttCTAGCaC** °
5	***+*4.7**	Int 2,+4,727	3	-	3	**GtcCTTGTTg** *CCA* **GGGCATGCCT**
6	+6.4	Int 3,+6,453	1	2	8	**GGtCTTGCTC**TGTTGCCC**AGGCTAGagT**
7	***+*6.8**	Int 3,+6,817	-	2	-	**AGGCTTGCCTcAGCATGTCg**
8	+14.3	Int 8,+14,269	2	1	2	**AGcCATGgTT**CC**AGcCAAGCCC**
9	+15.8	Int 9,+15,832	- in ¼ site	1	5	**TGTTT**GTTAG**AAGCATGTCa**

HS: half-site

* same RE described in [38].

° Cluster formed by 5 half-sites: the 3 central half-sites contain a CATG core and are separated by 0 bp spacer, while the two external half-sites are separated by the central 3 half-sites by 3 bp spacers. All of the 5 half-sites contain at least 2 mismatches each, although they never involve the core of the consensus.

Bold name: REs selected for experimental validation Bold/underlined: bases belonging to the indicated RE; italic: spacers; minuscule: mismatches within RE

Six REs were selected for validation, based on the evaluation of the following parameters: number and position of mismatches with respect to the consensus, presence of a spacer sequence between the two decameric sites, and position in the gene (Table 1). Three putative REs were excluded from further analysis: +6.4, which had an 8 bp spacer and mismatches in both decamers, involving in both cases a base near the core motif; +14.3, which had a 2 bp spacer and, again, mismatches close to the core in both decamers; and +15.8, which consisted of a three quarter site where the quarter site was separated from the half-site by a 5 bp spacer. Since we previously observed that the presence of mismatches and spacer length dramatically decrease RE functionality [37], and considering the distance from the TSS, these REs were not further investigated. Among the selected REs, two were located in the promoter of the *PRODH* gene, at positions -3.1 and -0.9 kb from TSS; the latter has already been described [38] and was investigated, although it did not completely fulfil the chosen parameters. The other REs were located in intron 2 (+1.7, +2.8 and +4.7 kb) and 3 (+6.8 kb), respectively. The latter RE, +6.8, falls within a genomic region previously identified in a ChIP -sequencing experiment but not further characterized [39]. In that paper, the RE was reported to be in intron 1, although according to a more recent genomic assembly and the *PRODH* mRNA version NM_016335.4 used in the present work, it is located in intron 3.

**Figure 2:**
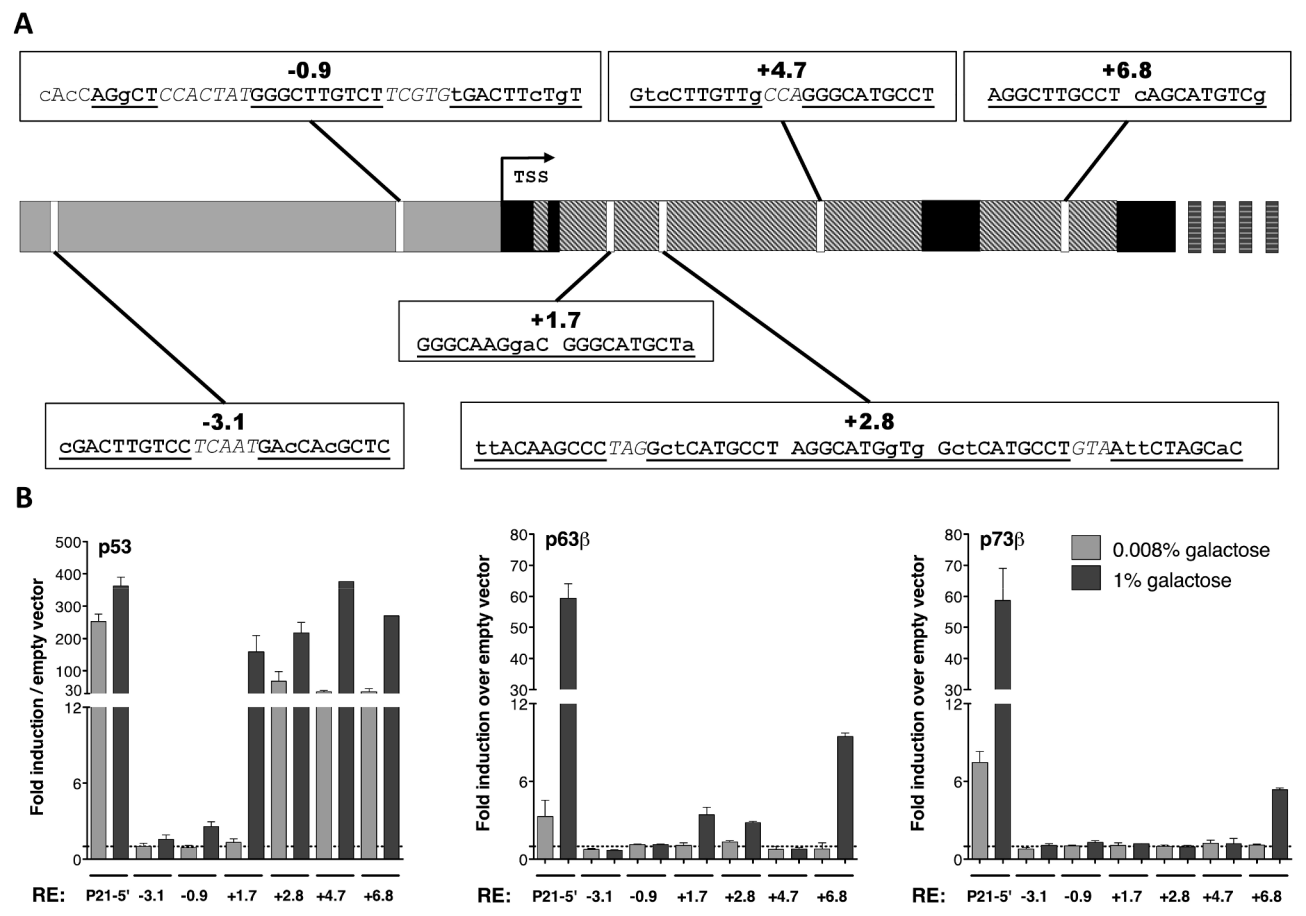
The *PRODH* gene contains several putative p53 REs, some of which are differentially transactivated by p53 family members in yeast. **A**. Scheme depicting chromosomal location and sequence of the six p53 REs in the *PRODH* gene that were selected for further analyses. The sequence of the REs at the various locations is shown (lowercase = mismatches from consensus; italics = spacer between half-sites) **B.** The REs in the *PRODH* gene were assayed with different p53 family members (p53, p63β and p73β) at different levels of galactose induction (0.008%, lighter bars and 1%, darker bars) in the yeast transactivation assay. As a positive control, the yeast strain carrying one of the p53 REs present in *P21* gene (P21-5’) was included. Bars represent the fold of induction obtained on each RE by the specified p53 family expression construct over the values obtained with the relative empty vector (negative control) (set to 1 and indicated by a broken line across each graph) used as negative control. The experiments shown derive from at least three biological replicates.

The two REs in the promoter contained only one half-site with either one (-3.1) or no (-0.9) mismatches (Figure 2A). An additional half-site with two mismatches, one of which involving a base in the CWWG core sequence, was present in the -3.1 RE, separated from the first half-site by a 5 bp spacer; in the -0.9 RE, a quarter site with one mismatch and a half-site with three mismatches (that can alternatively be considered a quarter site with one mismatch) were present, separated by the first half-site by 7 and 5 bp spacers, respectively (Figure 2A). Among the intronic REs, two (+1.7 and +6.8) had no spacers, while the +4.7 had a 3 bp spacer; the +4.7 and +6.8 REs had a consensus half-site and a second half-site with two mismatches outside the CWWG core motif, while the +1.7 had mismatches (one or two) in each half-site (Figure 2A). The +2.8 RE was identified as a full site composed of two half-sites (GctCATGCCT-AGGCATGgTg) by the TFbind software. Upon careful analysis of the nearby sequence, this RE turned out to be surrounded by other half-sites, thus constituting a cluster with a total of 5 half-sites, of which the 3 central contained a CATG core and no bp spacer, while the two external half-sites were separated from the central 3 REs by 3 bp spacers (Figure 2A). Each of the 5 half-sites in the +2.8 RE contained at least 2 mismatches, none involving the CWWG core motif of the consensus. Interestingly, the +6.8 was the only RE identified by the p53SCAN algorithm in the genomic region encompassing the *PRODH* gene [40].

### 3: The p53 family members differentially transactivate from the *PRODH* REs in yeast

Six yeast reporter strains, corresponding to the six selected REs in the *PRODH* gene (-3.1; -0.9; +1.7; +2.8; +4.7; +6.8, Figure 2A), were constructed using the “delitto perfetto” approach [30,41]. A well-established yeast transactivation assay was then applied to analyze the induction of the luciferase reporter gene upon expression of p53, p63β or p73β proteins. An isogenic yeast strain carrying the P21-5’ RE was used as positive control [32]. The results are presented as mean fold of induction over empty vector that was used as negative control. The expression of the p53 family members was regulated by using the galactose inducible promoter *GAL1,10*. Two different galactose concentrations were exploited to induce different levels of the transcription factors. The four intronic REs (+1.7, +2.8, +4.7 and +6.8) were induced by p53 in a dose dependent manner and showed strong response at the higher level of induction (1% galactose) (Figure 2B). At moderate induction (0.008% galactose), the +2.8, +4.7 and +6.8 already showed at least a 35-fold increase in luciferase activity above background. Overall, the levels of p53-dependent induction of the intronic REs were comparable to those obtained with the positive control strain, bearing the P21-5’ RE, which has been previously shown to be strongly induced by the p53 family members in yeast [32]. The REs in the promoter (-3.1 and -0.9) instead, showed no (-3.1) or extremely weak (2.5-fold, -0.9) induction over the basal level even at 1% galactose, suggesting lack of responsiveness to p53 (Figure 2B). Also the other p53 family members could weakly transactivate luciferase reporter expression from some REs, but only at 1% galactose. More specifically, the +1.7, +2.8 were very weakly transactivated upon p63β but not p73β expression, while the + 6.8 RE was transactivated following p63β or p73β expression (5 to 10-fold) (Figure 2B). At low galactose induction of p63β and p73β, no detectable increase in luciferase activity was observed in any of the *PRODH* REs (Figure 2B).

### 4: p73 transactivates *PRODH* in mammalian cells

**Figure 3:**
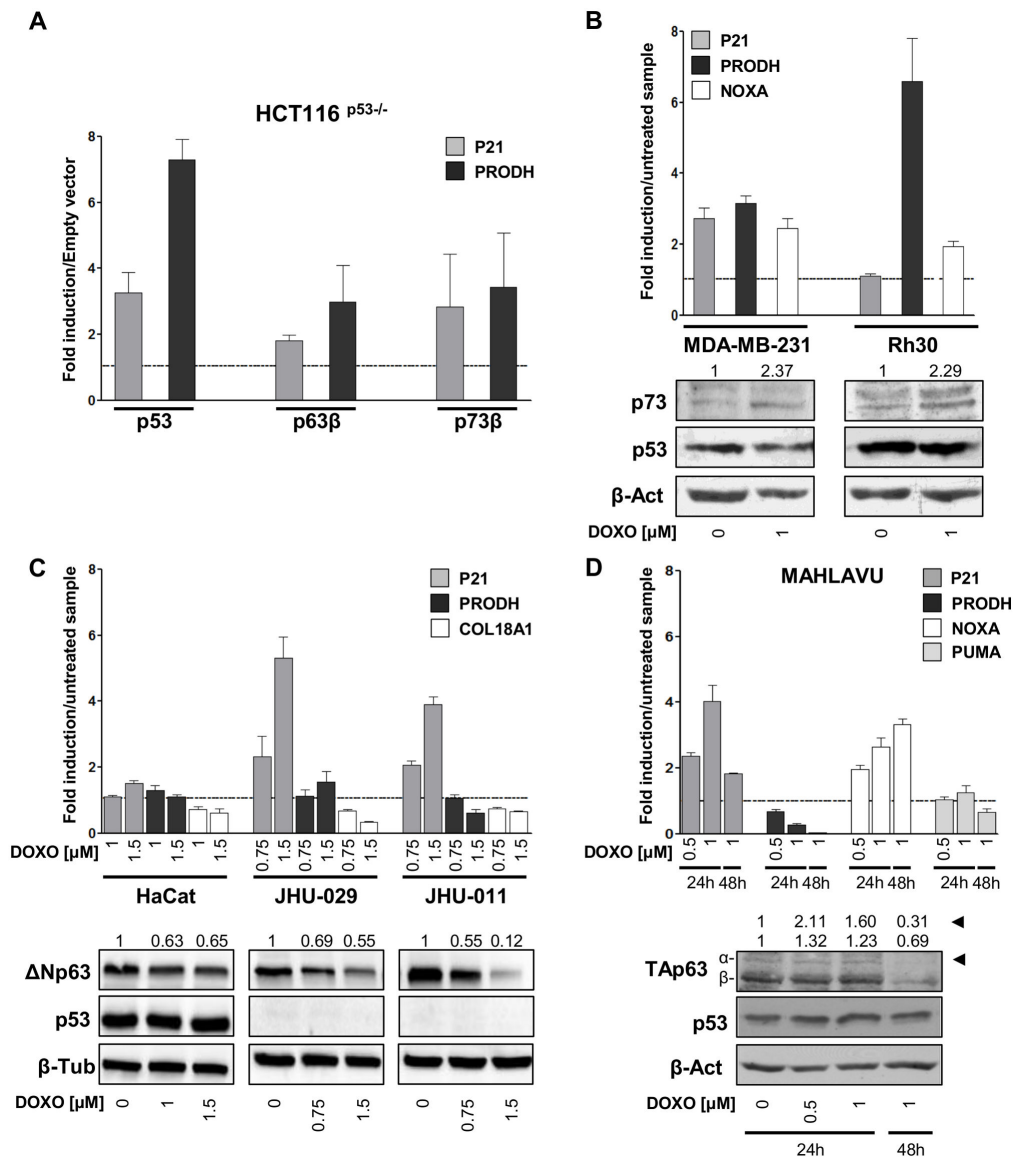
At least two p53 family members control the expression of *PRODH* in mammalian cells. **A**. Analysis of *PRODH* transcript levels after ectopic expression of p53 family members p53, p63β, p73β. Expression constructs for p53, p63β, p73β were transfected in HCT116 p53^-/-^ cells and *PRODH* transcript induction was assayed by qPCR and compared to the empty vector, used as reference and indicated by the broken line. **B**–**D**. Analysis of *PRODH* expression by qPCR in the indicated cell lines treated with DOXO and compared to untreated controls. Western blots of relevant proteins and of β-actin or β-tubulin used for normalization are shown. Variation of the relevant protein(s) in DOXO treated samples, compared to controls, after taking into account normalization with β-actin or β-tubulin, is reported above the western blots; for panel D, both TAp63 isoforms were quantified and the arrowheads indicate the TAp63α isoform and its quantification. **B**. Rh30 and MDA-MB-231 cell lines, expressing mutant p53 and TAp73β; **C**. cell lines with mutant (HaCat) or null (JHU-029, JHU-011) p53 and expression of ΔNp63α (HaCat, JHU-029, JHU-011); **D**. the Mahlavu hepatocarcinoma cell line, expressing mutant p53 and TAp63 isoforms. All the experiments shown are representative of at least three biological replicates.

To determine if p63 and p73 were capable of driving expression of the endogenous *PRODH* gene in mammalian cells, expression constructs for p53, p63β, p73β were transiently transfected into HCT116 p53^-/-^ cells. Cells transfected with empty vector represented the negative control and their value was arbitrarily set to 1 to calculate the fold of induction obtained with the p53 family members expression constructs. p63β and p73β expressing cells showed a 3-fold induction of PRODH, while p53 expressing cells showed a 7-fold induction (Figure 3A). Therefore, all members of the p53 family exhibit the potential to transactivate the endogenous *PRODH* gene in mammalian cells. To determine if *PRODH* was a physiological target of the whole p53 family we investigated *PRODH* expression resulting from genotoxic stress-induced modulation in cells lines that are mutant or null for p53 but express endogenous p63 or p73. Two cell lines, MDA-MB-231 and Rh30, were chosen to investigate the impact of p73 on *PRODH* expression. MDA-MB-231 is a breast carcinoma-derived cell line expressing the p53 p.R280K mutant. This mutant is considered to be loss-of-function based on the expression of physiological p53 targets and on functional assays in reconstituted systems (http://p53.fr/) [42]. The cells are reported to express TAp73β but no p63 [43]. Treatment with DOXO resulted in induction of the *PRODH* gene at levels comparable to *P21* and *NOXA*, used as positive controls (Figure 3B). The rhabdomyosarcoma-derived Rh30 cell line was also investigated for *PRODH* expression. This cell line expresses the p53 p. R280S mutant that is described as a loss-of-function allele as well (http://p53.fr/). The cells are reported to express TAp73β [43]. *PRODH* expression was induced by DOXO treatment at even higher levels than *NOXA* (Figure 3B). We were unable to see an induction of *P21* in this cell line at the conditions tested.

Three additional cell lines were instead used to explore *PRODH* responsiveness to endogenous p63: HaCat cells, derived from immortalized keratinocytes, and two cell lines derived from head and neck squamous cell carcinomas, namely JHU-029 and JHU-011. HaCat are compound heterozygous for two p53 mutations [H179Y and R282W, the former retaining residual transactivation function, while the latter being a loss-of-function allele (http://p53.fr/)]. These cells abundantly express ΔNp63α. JHU-029 and JHU-011 cells contain frameshift and nonsense p53 alleles, respectively, and do not express p53 proteins, based on western blot analysis (Figure 3C). Both cell lines express ΔNp63α; JHU-029 also expresses p73. Although ΔNp63α is not the most transactivation competent p63 isoform, it is commonly expressed in adult tissues as well as tumors, and specifically in squamous epithelial cells where is important for the regulation of specific target genes [44]. In all the three cell lines a reduction of p63 protein levels upon DOXO treatment was observed. mRNA expression analysis did not identify modulation of *PRODH* gene expression in HaCat cells. The reported ΔNp63α target gene *COL18A1* [45] was instead repressed, consistent with the reduction in ΔNp63α protein levels. *P21* appeared to be slightly induced, which could be related to a reduction of ΔNp63α-mediated repression [46] following the decrease in p63 protein levels after DOXO treatment. *PRODH* was differently regulated in JHU-029 and in JHU-011 cell lines: slightly induced in JHU-029, but repressed in JHU-011, consistent with the reduction of p63 protein (Figure 3C). Confirming previous reports [28], *P21* was inducible both in JHU-029 and in JHU-011, which could be p73-dependent. *COL18A1* was instead repressed in both cell lines, paralleling the reduced level of p63 protein. Taken together, our results are supporting p73-mediated induction of *PRODH*, while the data obtained with the cell lines expressing ΔNp63α are less conclusive and univocal.

Finally, *PRODH* expression was analyzed in the Mahlavu hepatocellular carcinoma cell line after DOXO treatment. Mahlavu cells express the p53 p. R249S mutation, that retains very low residual activity based on functional assays in reconstituted systems (http://p53.fr/). These cells were reported to constitutively express TAp63α. Importantly, after treatment with DNA damaging agents all TAp63 isoforms were reported to be induced, although only a limited number of targets was shown to be subsequently activated, resulting preferentially in G2/M arrest [47]. In our experiments, based on western blotting, both TAp63α and TAp63β were expressed in Mahlavu cells and both were only slightly induced by DOXO (Figure 3D). While *P21* was induced after DOXO in this cell line, as previously reported [47], and so was *NOXA*, we were unable to see any induction of either *PRODH* or *PUMA* (Figure 3D). This differential target expression may be due to a residual, selective activity of the specific p53 mutant expressed in this cell line or may be cell type or cell line specific. More TAp63 expressing cell lines, possibly deriving from other cell types, should be tested before drawing any definitive conclusions on endogenous p63 mediated *PRODH* transactivation.

### 5. The +6.8 RE shows the highest p53 binding *in vivo*

To study p53 binding to the *PRODH* gene *in vivo*, HCT116 p53^+/+^ cell line and its p53 knockout derivative (HCT116 p53^-/-^), were treated with DOXO or left untreated (mock) and cell extracts were subjected to ChIP analysis.

**Figure 4:**
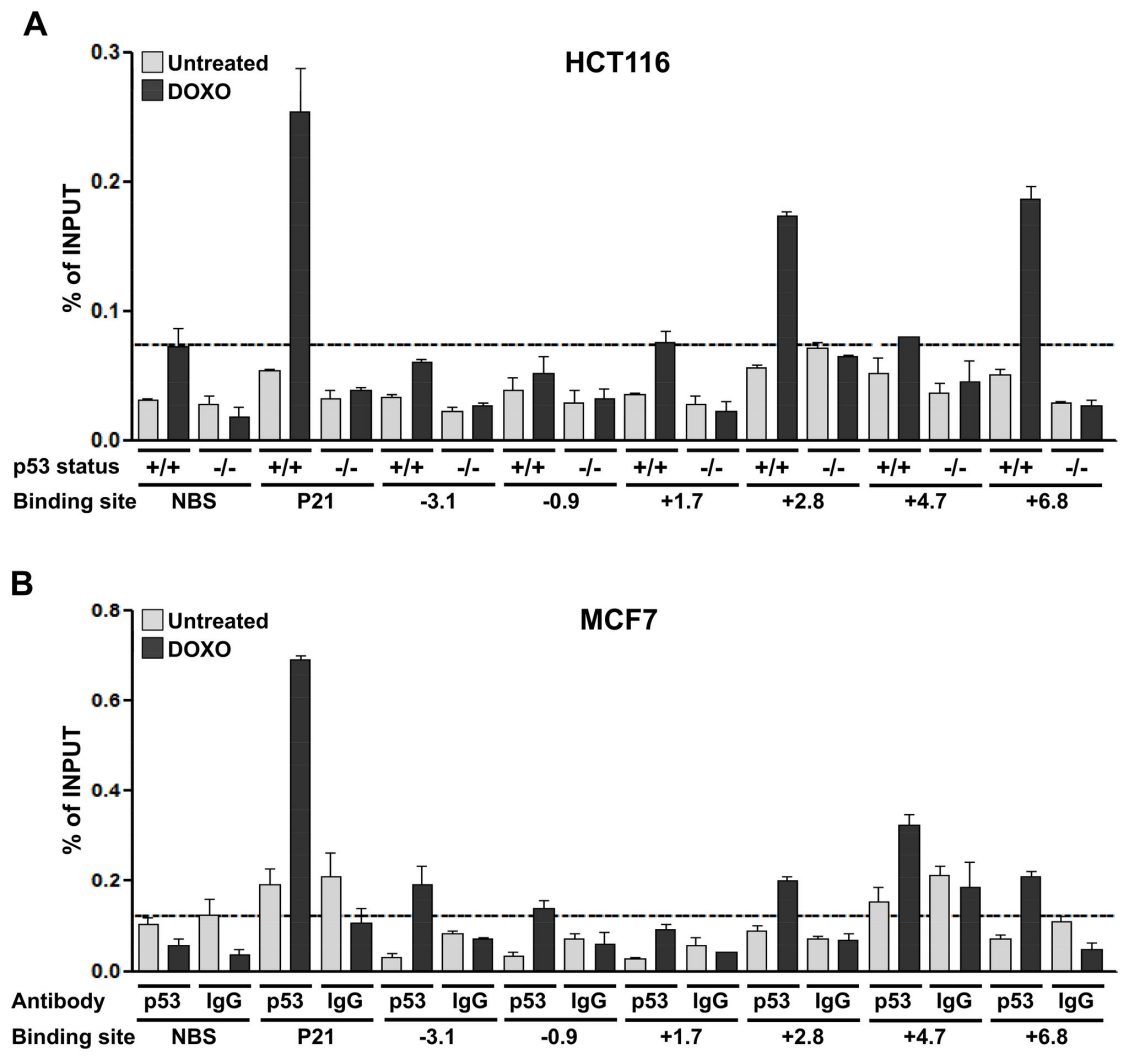
Relative p53 occupancy levels at *PRODH* sites containing p53 REs. ChIP-qPCR experiments were performed in: **A**. HCT116 p53^+/+^ cell line and its isogenic derivative HCT116 p53^-/-^ (negative control), treated with DOXO or left untreated. The broken line indicates the level of p53 bound to the No Binding Site (NBS) after DOXO treatment in the HCT116 p53^+/+^ cells; **B**. the MCF7 cell line, treated with DOXO or left untreated. As a control for aspecific antibody binding, immunoprecipitation with IgG was also performed. The broken line indicates the enrichment of IgG immunoprecipitated material over the input, that represents the highest value obtained for the NBS negative control. The graphs show the binding of p53 to the genomic regions surrounding the REs in the *PRODH* gene, as well as the binding to a genomic region not containing a p53 RE (NBS), used as negative control, and to the genomic region containing the *P21-5*’ RE (positive control). Data shown are representative of two independent experiments and the values obtained are expressed as percentage of input.

The data showed that the +2.8 and the +6.8 REs were the sequences most efficiently bound by p53 in HCT116 p53^+/+^ after DOXO treatment, showing a 2-fold or higher enrichment, respectively, when compared to the p53 immunoprecipitated material in mock conditions and in the DOXO treated HCT116 p53^-/-^ cells (Figure 4A). To determine specificity of p53 enrichment at each RE after DOXO treatment we also applied a cutoff, choosing the highest value obtained for binding of p53 at a genomic region not containing a p53 RE (an average of the signal with beta-actin and *CCNB1* genes, indicated as No Binding Site, NBS) (Figure 4A). Given the high level of p53 bound to the NBS site, the +1.7 and +4.7 REs, although enriched in bound p53 after DOXO when compared to the other conditions at the same locus, could not be considered as positive (Figure 4A).

To verify whether p53 binding to *PRODH* regulatory elements was a general and physiological response to DNA damage, we repeated ChIP experiments in the breast adenocarcinoma cell line MCF7, expressing wild-type p53. To control for specificity of p53 binding, IgG was used and the binding of DO-1 or IgG to the specific REs or the NBS in stressed (DOXO) or mock conditions was compared. PRODH -3.1, +2.8, +4.7 (despite a high background in control conditions as well), +6.8 and, at a minor extent, -0.9 REs were enriched in p53 immunoprecipitated material after DOXO treatment, when compared to IgG immunoprecipitated controls in the same conditions, and to DO-1 immunoprecipitation in mock conditions. p53 binding to *PRODH* REs was confirmed also when a cutoff, based on binding of IgGs to NBS, was applied.

In conclusion, our results suggest that two (+2.8 and +6.8 in HCT116 p53^+/+^) or more (-3.1, -0.9, +2.8, +4.7, +6.8 in MCF7) of the p53 REs identified in the *PRODH* gene are indeed bound *in vivo* by p53 and that binding increases upon DNA damage.

### 6: p53, but not p63 and p73, weakly transactivates from the *PRODH2* REs in yeast

**Figure 5:**
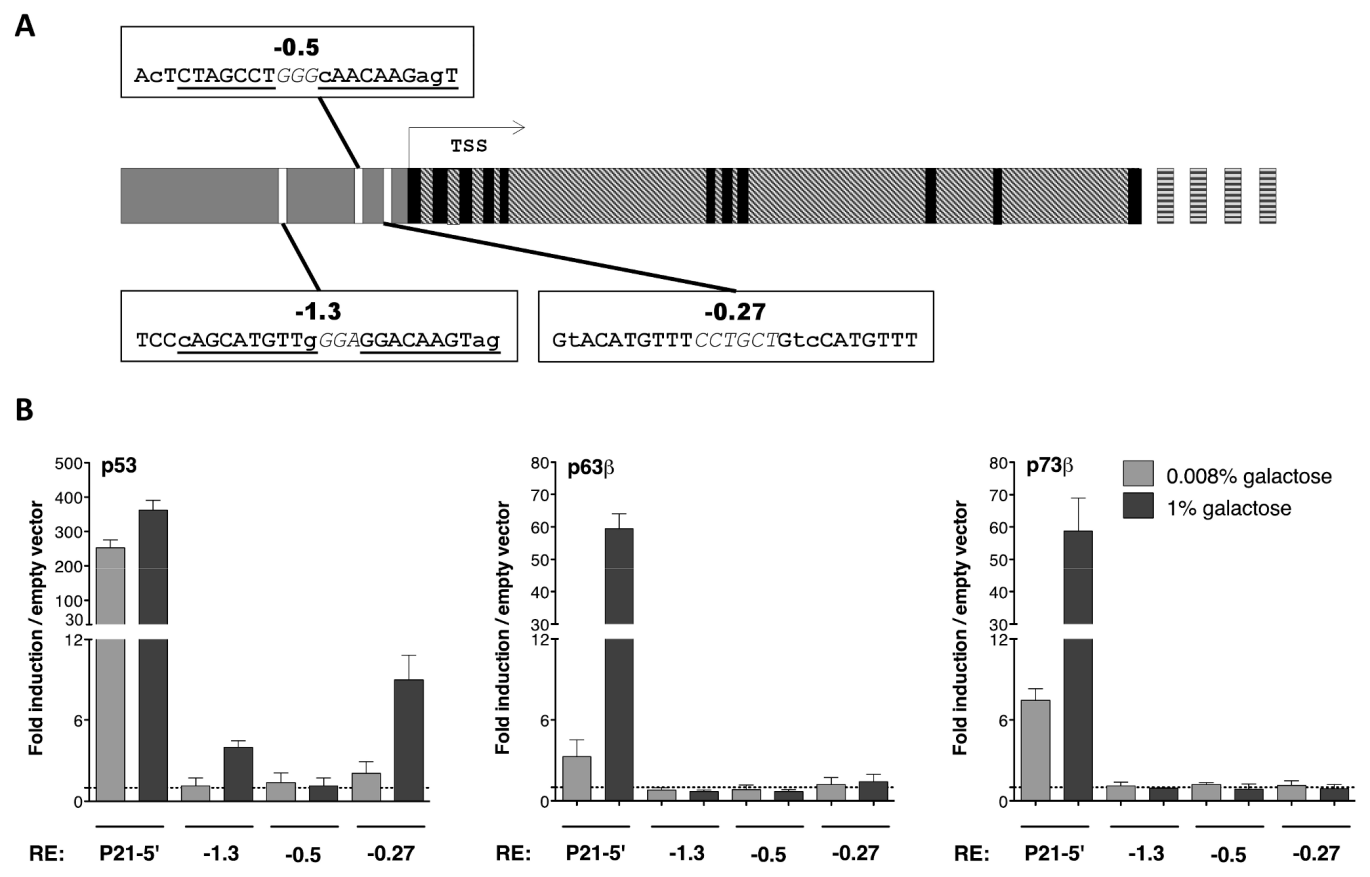
The *PRODH2* gene contains three putative p53 REs, two of which are poorly transactivated only by p53 in yeast. **A**. Scheme depicting chromosomal location and sequence of the p53 REs in the *PRODH2* gene, selected for analysis in the yeast transactivation assay. **B**. The REs in the *PRODH2* gene were assayed with different p53 family members (p53, p63β and p73β) at different levels of galactose induction (0.008%, lighter bars and 1%, darker bars) in the yeast transactivation assay. As a positive control, the yeast strain carrying the *P21-5*’ RE was included. Bars represent the fold of induction of each RE by the specified p53 family expression construct over the values obtained with the relative empty vector (negative control) (set to 1 and indicated by a broken line across each graph). All the experiments derive from at least three biological replicates.

Five putative p53 REs were identified in the *PRODH2* gene, three of which were in the promoter, while two were located in intron 9 at more than 10 kb from the TSS (first nucleotide present in the NM_021232.1 reference *PRODH2* mRNA) (Table 2). The latter two REs consisted of just one half-site (Table 2); for this reason and for the distance from the TSS, they were excluded from further analysis. Of the three REs identified in the promoter (Table 2 and Figure 5A), two had a 3 bp spacer and at least four mismatches in the two half-sites, not involving the core sequence (-1.3 and -0.5), and the third (-0.27) had a 6 bp spacer and one or two mismatches in the two half-sites (Table 2 and Figure 5A).

**Table 2:** p53 Response elements in the *PRODH2* gene.

	Name	Location (bp from TSS)	Mismatches HS 1	Mismatches HS 2	Spacer (bp)	Sequence
1	**-1.3**	Promoter, -1,281	2	2	3	**cAGCATGTTg** *GGA* **GGACAAGTag**
2	**-0.5**	Promoter, -0,534	2	3	3	**ActCTAGCCT** *GGG* **cAACAAGagT**
3	**-0.27**	Promoter, -0,267	1	2	6	**GtACATGTTT** *CCTGCT* **GtcCATGTTT**
4	+10.5	Intron 9,+10,519	2	NA	NA	**cAGCAAGaCC**
	5	+10.7	Intron 9,+10,685	NA	NA	**AAGCAAGTCC**

HS: half-site Bold: REs selected for experimental validation Bold/underlined: bases that are part of the indicated RE; italic: spacers; minuscule: mismatches within RE

Yeast strains carrying the -1.3, -0.5 and -0.27 REs upstream of the chromosomally located luciferase reporter, and otherwise isogenic with the previously described *PRODH* RE strains, were constructed. Again, an isogenic strain carrying the *P21-5*’ RE was used as positive control. Activity of the reporter was only slightly increased by high-level p53 expression in the three PRODH2 strains, with -0.27 being the most efficiently transactivated (9-fold induction) (Figure 5B). A 4-fold increase in luciferase activity over the empty vectors, used as negative control, was obtained with the -1.3 RE but only a 2-fold with the -0.5 RE. Expression of p63ß and p73ß did not result in any detectable induction of luciferase activity (Figure 5B). Taken together the results suggested a weak responsiveness of *PRODH2* gene to p53 and no responsiveness to p63 or p73.

### 7: p53 weakly transactivates the *PRODH2* gene in mammalian cells

**Figure 6:**
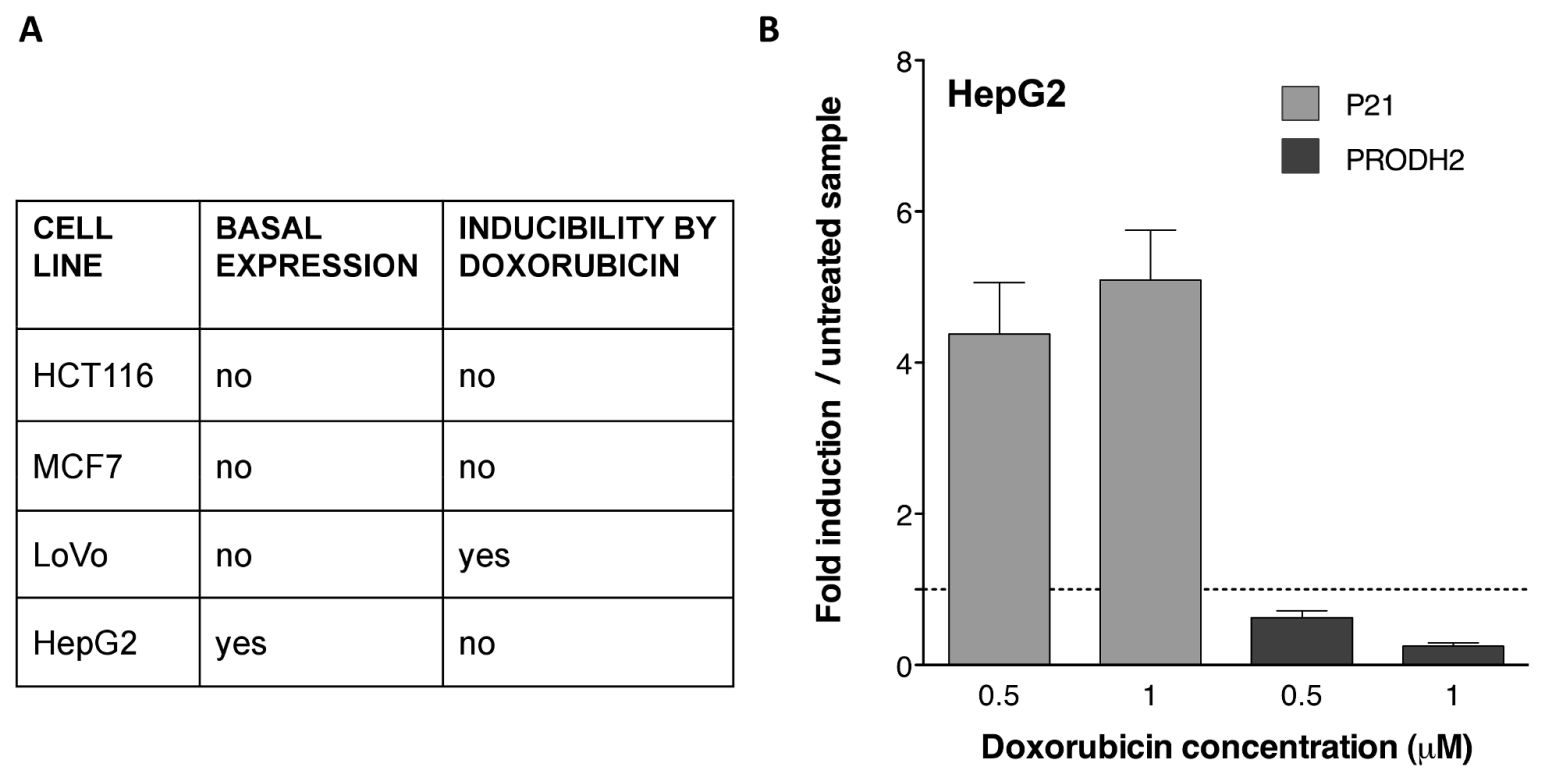
p53 weakly induces *PRODH2* expression in some cell lines. **A**. The ability of DOXO to induce expression of the *PRODH2* gene was analysed in four cell lines harbouring endogenous wild-type p53, namely HCT116 (p53^+/+^), MCF7, LoVo and HepG2. **B**. Levels of *PRODH2* (darker bars) and *P21* (lighter bars, positive control) transcripts in HepG2 cells treated with DOXO were determined by qPCR. The value obtained in the untreated cell line was normalized to 1 (broken line). All the experiments were performed in triplicate.

To verify if *PRODH2* is inducible by genotoxic stress via p53, we treated different cell lines known to harbour wild-type p53 with DOXO or 5-FU, as previously described for induction of *PRODH*. Consistent with the literature, these genotoxic treatments resulted in a slight increase of *PRODH2* transcript in LoVo cells, but we were unable to calculate the fold induction as basal levels fell below the detection limits of our qPCR (Figure 6A). In HCT116 p53^+/+^ and MCF7 cell lines, basal levels were undetectable and no induction was observed (Figure 6A). Finally, when the HepG2 hepatocarcinoma cell line, which turned out to have detectable basal levels of *PRODH2*, was treated with DOXO, no induction of the *PRODH2* gene was observed (Figure 6B). Concomitantly, a slight repression was detected, in spite of efficient induction of the *P21* transcript, used as a positive control.

Taken together, and compared with the induction levels obtained with *PRODH*, *PRODH2* should be considered as a weak p53 target with low expression levels and limited p53 responsiveness in human cells.

## Discussion

In the present study, the identification of putative p53 REs in the *PRODH* and *PRODH2* genes and the experimental analysis of their functionality are described. Indeed, these genes have been previously described as p53 targets [15,19,38], although the REs were not characterized. They encode proteins involved in similar but not identical metabolic processes in the cell. In fact, their catalytic activity is directed on very similar substrates of common origin (dietary protein or collagen degradation) with limited cross-reactivity with each other [48]. Furthermore, both proteins are capable of inducing apoptosis via ROS production [16,19].

In spite of the fact that PRODH has been known for a long time as a p53 pro-apoptotic effector [15], there is very limited information on the regulatory elements that mediate p53 responsiveness of the corresponding gene. This could explain why *PRODH* was not included in the list of 129 genes responding to at least three out of four of the criteria -namely the presence of a p53 RE, up-regulation by wild-type p53, responsiveness of the identified RE in functional assays and physical binding of RE by p53-to be classified as a p53 regulated gene [2,49].

Here, we extended the characterization of *PRODH* responsiveness to p53 beyond genotoxic induction, by showing that it was also strongly induced by p53 stabilization following Nutlin-3A treatment (Figure 1). We also showed, by use of a yeast transactivation assay, that p53 exhibits transactivation potential towards all intronic REs identified in the *PRODH* gene (Figure 2B). The intronic REs presented at least two complete half-sites and no mismatches in the core sequences, in contrast to the REs present in the promoter (Table 1). Ultimately, the extent of p53-dependent transactivation of the *PRODH* gene may be due to the sum of the contribution of each functional RE. Moreover, depending on p53 levels one might expect titration of p53 to the REs based on its affinity resulting in different levels of *PRODH* induction, which may influence its activity [17,50,51]. Finally, we showed that some of the REs identified are directly bound by p53 *in vivo*.

The only data available in the literature before the present work reported on a putative p53 binding site in the *PRODH* promoter, for which weak p53-dependent transactivation was shown to be associated with p53 binding [38]. The observed transactivation based on ectopic p53 overexpression in a p53-null cell line was very low, less than 2-fold in the first 48 hours and reached only 3.5-fold 72 hours after transfection [38]. Taking into account the current genome annotation and position of TSS, this binding site, previously located at position -1.161 bp, corresponds to the -0.9kb RE (present work), shown to be inactive in the transactivation assays and poorly bound by p53 in ChIP analyses in two cell lines. The putative RE contains a consensus half-site with a rigid, lower affinity CTTG core motif [52,53], and an additional half-site, separated by a 5 bp spacer that also contains two mismatches in critical positions. Based on previous studies on the *cis*-element requirements for p53-induced transactivation, we would predict this RE to behave as a single half-site with very low transactivation potential [37,54]. The very low activity observed by Maxwell and Kochevar [38] is indeed compatible with the responsiveness reported for a single p53 half-site [37,55]. In the yeast-based assay we used, ectopic p53 expression is controlled by an inducible promoter and the results obtained at different levels of expression strongly suggest that the putative RE in the *PRODH* promoter region is, at best, very weakly functional (see Figure 2 panel A). In conclusion, *PRODH* modulation by p53 seems to be mainly due to the other REs we identified (Figure 2B and Figure 4). Among these, the +6.8 was the RE most efficiently transactivated by all members of the p53 family and is embedded in a genomic region well represented in several genome wide p53 ChIP analyses [39,40] (D. Menendez and M. Resnick, personal communication). The genomic region containing the +6.8 RE, together with the +2.8 RE, was recently reported to be bound by p53 in the MCF7 cell line, after various treatments [56]. We found that the same regions were enriched in p53 immunoprecipitated material in both DOXO treated MCF7 and HCT116 p53^+/+^ cell lines. In our experimental settings, however, other REs, among those identified, were bound by p53 in the MCF7 cell line. These results were mostly consistent with the ones obtained in the yeast functional assays, confirming the reliability of the data.

Concerning *PRODH2*, our results could not conclusively demonstrate its responsiveness to p53: in fact, the experimental results suggest that it is at best a very weak p53 target gene. This conclusion is based on the functional analysis of identified p53 REs in yeast (compare Figures 2 and 5) and on the quantification of the endogenous gene expression in different cell lines upon genotoxic stress-dependent induction of p53 (Figure 6). The latter analysis was limited by the undetectable basal level of *PRODH2* expression in most of the cell lines that were examined (i.e. HCT116, MCF7, LoVo). However, in the HepG2 cell line, where a basal level was clearly detectable, no induction of *PRODH2* by activation of p53 was observed. Recently, the group led by Phang demonstrated *PRODH2* induction by p53 both in RKO and LoVo cell lines, although basal expression was only observed in RKO colon cancer cell line [19]. In agreement with their findings, in the LoVo cell line we could confirm absence of basal expression but its induction upon genotoxic stress, that was however at the limit of detection by qPCR. Shinmen et al. [20] could not detect any induction of *PRODH2* upon p53 transfection or stabilization by Nutlin in the U87 glioblastoma-derived cell line, similar to what we observed in HepG2 cells. From all these observations, it appears that both *PRODH2* basal expression and its induction by p53 are cell line-dependent (Table S3 and Figure 6). It is possible that transcription of certain targets by p53 is influenced by the presence of other transcriptional co-factors in these cell lines [20].

Interestingly, Nikulenkov et al. found that, after 5FU and Nutlin3A treatment, p53 weakly bound to a *PRODH2* genomic region [56]. The level of binding was considerably lower than *PRODH* or other p53 common targets. The genomic region differed in the two treatments and mapped about 6.8 and 15.5 kb upstream of the *PRODH2* TSS, respectively, a region that lied outside the genomic range we considered in our analysis, that started 5kb upstream of TSS. Nevertheless, there is no evidence that these regions actually control the expression of *PRODH2*. In our analyses we did not find *PRODH2* induction in the MCF7 cell line upon genotoxic damage (Figure 6).

In conclusion, although a coordinated expression of PRODH and PRODH2 would be justified by the existence of proteins, like collagen, rich both in proline and OH-proline, respectively the substrates of the two enzymes, this does not seem to be the case [57]. Indeed, PRODH has a broad pattern of expression and could contribute to cell metabolism by the production of glutamate and α-KG from P5C, compounds in turn involved in many metabolic reactions and pathways in the cell.

On the lack of a coordinated p53-dependent expression of PRODH and PRODH2 some considerations can be taken into account. First, and notably, the step downstream of the PRODH reaction in the pathway leading from proline to glutamate is catalyzed by P5C dehydrogenase, whose gene (*ALDH4*) was reported as a p53 target [18]. Second, other p53 transcriptional targets, such as TIGAR [58,59], can modulate α-KG levels. This suggests an important, and only partially elucidated, contribution of the latter compound in p53 mediated responses and stresses the contribution of PRODH in the metabolic pathways controlled by p53 [4].

Finally, in this work we also addressed the responsiveness of the *PRODH* gene to other members of the p53 family. Indeed, we found that ectopic expression of p63β and p73β could also induce *PRODH*, even though at lower levels compared to p53 (Figure 2A). We also showed that induction of endogenous TAp73β by DOXO in two cell lines (Rh30 and MDA-MB-231) leads to an increase in *PRODH* transcript (Figure 2B), while data obtained for p63 were less clear-cut, probably because of the p53 family members endogenously expressed and their cross talk. Indeed, three of the cell lines we chose for analyzing the contribution of p63 to *PRODH* transactivation (JHU-011; JHU-029; HaCat) express mainly the ΔNp63α isoform. This isoform has been shown to be able to transactivate or repress different subsets of p53 responsive genes as well as a set of peculiar genes [7,44] and is the p63 isoform commonly expressed in adult tissues, tumors and tumor derived cell lines [44]. Interestingly, expression of the ΔNp63α isoform in the yeast strain carrying the +6.8 RE was capable of driving luciferase activity in the transactivation assay (unpublished result). Finally, we found that in the Mahlavu hepatocellular carcinoma cell line *PRODH* expression was diminished upon DOXO treatment. The isoforms expressed, that were found only slightly induced by DOXO in this cell line, are TAp63α and TAp63β (Figure 3D), the first of which does not transactivate the *PRODH* REs in the yeast assay (unpublished results). We do not have an explanation for this observation, except interaction between the different isoforms could impact on their transactivation ability. More cell lines expressing TAp63 will need to be examined before drawing any conclusions on transactivation of *PRODH* by endogenous p63.

To our knowledge, no data are available on p73 ability to transcriptionally regulate *PRODH* expression, while *PRODH* had been previously found as one of the genes expressed more than 4-fold upon expression of a tetracycline-inducible TAp63γ isoform [60]. The lower levels of induction with respect to p53 were not unexpected, as several other p53 targets show a decreased responsiveness to p63 and p73. Furthermore, this result could be explained, among others, by the fact that p63 and p73 show somewhat different DNA binding affinity and transactivation potential towards canonical p53 REs, possibly in part dependent on tetramer assembly and conformation stability, as recently revealed by the comparison of crystal structures of p63 and p73 bound to DNA [61–64]. It is interesting to note that the three REs transactivated by p63 (namely +1.7, +2.8 and +6.8) and the one responsive to p73 (+6.8) all have at least two half-sites with no spacer, consistent with previous studies indicating both for p63 and p73 a marked preference for adjacent half-site REs for both p63 and p73 [14,63,65]. The +6.8kb RE turned out to be the most efficiently recognized not only by p53 but also by p63 and p73. The reason for this may be that this RE has no spacer and that the mismatches present in one half-site affect the first and last base of the consensus, which are not involved in establishing direct protein::DNA interactions and does not preclude high affinity binding of p53 [49,66–68]. However, since in mammalian cells p63 and p73 transactivate equally the *PRODH* gene in transient transfection assay, induction appears not to be strictly correlated to the relative transactivation potentials measured in the yeast-based assay. Notably, also p73, besides p53, has been recently implicated in regulation of metabolism and autophagy and has been shown to be regulated by mTOR [43,69,70]. As proline dehydrogenase is induced by rapamycin [71], it is tempting to speculate that this may be achieved at least in part through p73.

In conclusion, this work demonstrates that *PRODH* is a target of at least two p53 family members and provides new clues for a deeper involvement of p53 proteins in metabolic pathways. In fact, in light of the recently described link between glutamine and proline, p53 acquires a more profound role in metabolism of these non essential amino acids and their derivative α-KG and in antagonizing c-Myc, that was recently found to downregulate *PRODH* as an important contribution to metabolic reprogramming and induction of cell proliferation [72–75].

## Supporting Information

Table S1 Oligonucleotides used as primers in the present work.
(DOCX)

Table S2 Oligonucleotides used for the creation of PRODH and PRODH2 yeast reporter strains by the “delitto perfetto” approach.
(DOCX)

Table S3 Summary of the cell lines analysed for *PRODH2* induction by p53 in the present and previous studies.

Information S1 Letter from Michael A. Resnick, giving authorization to cite unpublished data as personal communication.
(DOC)
