# Supplementary material for: P53 Family Members Modulate the Expression of PRODH, but Not PRODH2, via Intronic p53 Response Elements
Source: PLoS One. 2013 Jul 8;8(7):e69152. doi: 10.1371/journal.pone.0069152 (PMC3704516; doi:10.1371/journal.pone.0069152)
Supplement: Table S1 — Oligonucleotides used as primers in the present work. (DOCX) [file pone.0069152.s001.docx]

Table S1. Oligonucleotides used as primers in the present work

| **NAME** | **SEQUENCE (**5’ - 3’ orientation) | **USE** |
| --- | --- | --- |
| **ade2_Fw** | aagttgcctagtttcatgaa | Yeast colony PCR and |
| **luc1_Rv** | catagcttctgccaaccgaa | Sequencing after “*delitto perfetto*” |
|  |  |  |
| **PIG6_prom-3.1_F** | tgccccattatcaccctagcttct | qPCR after ChIP |
| **PIG6_prom-3.1_R** | tgcccaacatggaggcttttct |  |
| **PIG6_prom-0.9_F** | atactcaccaggctccactatggg |  |
| **PIG6_prom-0.9_R** | cggccacaagttgtatggttcgtt |  |
| **PIG6_int2+1.7_F** | ccacatctaaggggcatcccaaaa |  |
| **PIG6_int2+1.7_R** | cacaaggtgggcatggcttct |  |
| **PIG6_int2+2.8_F** | tggttgctttgctgtggagtca |  |
| **PIG6_int2+2.8_R** | agacagggtttcacgatgttgctc |  |
| **PIG6_int2+4.7_F** | ctgtggactgtcatctagctca |  |
| **PIG6_int2+4.7_R** | tgttccctcttatcccaagtcc |  |
| **PIG6_int3+6.8_F** | aaggggagggaaaggcagtca |  |
| **PIG6_int3+6.8_R** | caaaacagccaatcgcaaggca |  |
| **CCNB1_FW (NBS)** | tatgccacatcgaagcatgctaa |  |
| **CCNB1_RV (NBS)** | acagatggcacatggtgccaatt |  |
| **P21-5’_F ChIP** | gtggctctgattggctttctg |  |
| **P21-5’_R ChIP** | ctcctaccatccccttcctc |  |
|  |  |  |
| **PRODH FW sybr** | cagccacatggagacattcttg | qPCR |
| **PRODH RV sybr** | agccgtcatcgctgactctac |  |
| **P21 FW sybr** | ctggagactctcagggtcgaaa |  |
| **P21 RV sybr** | gattagggcttcctcttggagaa |  |
| **Beta-2MG FW sybr** | aggctatccagcgtactcca |  |
| **Beta-2MG RV sybr** | atggatgaaacccagacaca |  |
| **GAPDH FW sybr** | gaaggtgaaggtcggagtc |  |
| **GAPDH RV sybr** | gaagatggtgatgggatttc |  |
| **NOXA FW sybr** | agctggaagtcgagtgtgct |  |
| **NOXA RV sybr** | tcctgagcagaagagtttgga |  |
| **PUMA FW sybr** | cctggagggtcctgtacaatct |  |
| **PUMA RV sybr** | gcacctaattgggctccatct |  |
| **COL18A1 FW sybr** | accacctcttcagagcgatcag |  |
| **COL18A1 RV sybr** | cgacgtcggggtcatccgtct |  |
|  |  |  |
| **PRODH** | Hs00271933_m1 | AB Taqman assays |
| **PRODH2** | Hs00560403_m1 |  |
| **Beta-2MG** | Hs00984230_m1 |  |
