# Supplementary material for: P53 Family Members Modulate the Expression of PRODH, but Not PRODH2, via Intronic p53 Response Elements
Source: PLoS One. 2013 Jul 8;8(7):e69152. doi: 10.1371/journal.pone.0069152 (PMC3704516; doi:10.1371/journal.pone.0069152)
Supplement: Table S2 — Oligonucleotides used for the creation of PRODH and PRODH2 yeast reporter strains by the “delitto perfetto” approach. (DOCX) [file pone.0069152.s002.docx]

Table S2. Oligonucleotides used for the creation of PRODH and PRODH2 yeast reporter strains by the “*delitto perfetto*” approach

| **Name** | **Sequence of the Response Element (RE)** |
| --- | --- |
| **PRODH -3.1** | **cGACTTGTCC***TCAAT***GAcCAcGCTC** (25)* |
| **PRODH -0.9** | cAcC**AGgCT***CCACTAT***GGGCTTGTCT***TCGTG***tGACTTcTgT** (41) |
| **PRODH +1.7** | **GGGCAAGgaCGGGCATGCTa** (20) |
| **PRODH +2.8** | **ttACAAGCCC***TAG***GctCATGCCTAGGCATGgTgGctCATGCCT***GTA***AttCTAGCaC** (56) |
| **PRODH +4.7** | **GtcCTTGTTg***CCA***GGGCATGCCT** (23) |
| **PRODH +6.8** | **AGGCTTGCCTcAGCATGTCg** (20) |
| **PRODH2 -1.3** | **TCCcAGCATGTTg***GGA***GGACAAGTag** (26) |
| **PRODH2 -0.5** | **AcTCTAGCCT***GGG***cAACAAGagT** (23) |
| **PRODH2 -0.27** | **GtACATGTTT***CCTGCT***GtcCATGTTT** (26) |

* Number in brackets indicates the length of the Response Element in nucleotides.

In the complete oligonucleotide used for “*delitto perfetto*” the sequence of each Response Element is surrounded at both sides by the sequences needed for homologous recombination in yeast; these sequences are:

**5’ CORE** GCGGAATTGACTTTTTCTTGAATAATACAT (30)

**3’ CORE** GCAGATCCGCCAGGCGTGTATATAGCGTGG (30)

the total length of each oligonucleotide is the sum of 5’core+ RE+3’ core.
