# Supplementary material for: P53 Family Members Modulate the Expression of PRODH, but Not PRODH2, via Intronic p53 Response Elements
Source: PLoS One. 2013 Jul 8;8(7):e69152. doi: 10.1371/journal.pone.0069152 (PMC3704516; doi:10.1371/journal.pone.0069152)
Supplement: Table S3 — Summary of the cell lines analysed for PRODH2 induction by p53 in the present and previous studies. [file pone.0069152.s003.docx]

Table S3. Summary of the cell lines analysed for PRODH2 induction by p53 in the present and previous studies.

| Cell line | Origin | p53 status | PRODH2 transcript -basal expression | PRODH2 transcript -induction by DOXO/p53 | Reference |
| --- | --- | --- | --- | --- | --- |
| MCF7 | breast | wild-type | NO | NO* | Present study |
| HepG2 | liver | wild-type | YES | NO | Present study |
| HCT116 | colon | wild-type | NO | NO* | Present study |
| LoVo | colon | wild-type | NO | YES | Present study and [19] |
| RKO | colon | wild-type | YES | YES | [19] |
| HT29 | colon | mutant | N.A. | Not available ** | [19] |
| HCT15 | colon | mutant | N.A. | Not available ** | [19] |
| U87 | glia | wild-type | NO | NO | [20] |

* Not detected under the qPCR conditions tested

** Only enzyme activity was tested in these cell lines. The authors showed an increase in activity upon p53 transfection.
