# Supplementary material for: P53 Family Members Modulate the Expression of PRODH, but Not PRODH2, via Intronic p53 Response Elements
Source: PLoS One. 2013 Jul 8;8(7):e69152. doi: 10.1371/journal.pone.0069152 (PMC3704516; doi:10.1371/journal.pone.0069152)
Supplement: Information S1 — Letter from Michael A. Resnick, giving authorization to cite unpublished data as personal communication. (DOC) [file pone.0069152.s004.doc]

|  | DEPARTMENT OF HEALTH & HUMAN SERVICES Public Health Service |
| --- | --- |

National Institutes of Health

National Institute of

Environmental Health Sciences

111 Alexander Dr.

P. O. Box 12233

Research Triangle Park, NC 27709

FAX: 919 541-7593 PH: 919 541-4480

E-mail: resnick@niehs.nih.gov

July 8, 2012

PLoS One

To whom it may concern:

I, the undersigned, hereby authorize Dr. Campomenosi to cite as “personal communication” unpublished data related to in silico as well as experimental validation of a p53 binding site in the region of the PRODH gene regulatory region derived from our ChIPseq experiments in which U2OS cells were treated with Doxorubicin and Nutlin.

Sincerely,


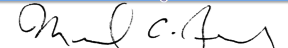


Michael A Resnick

Head, Chromosome Stability Group

NIEHS, NIH
